# Association of CD44 polymorphisms and susceptibility to HBV‐related hepatocellular carcinoma in the Chinese population

**DOI:** 10.1002/jcla.22977

**Published:** 2019-07-13

**Authors:** Yan Deng, Zhi‐jian Chen, Fang Lan, Qi‐tian He, Si‐yuan Chen, Yu‐fang Du, Shan Li, Xue Qin

**Affiliations:** ^1^ Department of Clinical Laboratory First Affiliated Hospital of Guangxi Medical University Nanning Guangxi China; ^2^ Department of Clinical Laboratory Guangxi Maternal and Child Health Hospital Nanning Guangxi China; ^3^ Department of Clinical Laboratory Second Affiliated Hospital of Guangxi Medical University Nanning Guangxi China

**Keywords:** CD44, hepatocellular carcinoma, polymorphism

## Abstract

**Background:**

This study aimed to determine whether CD44 polymorphisms were correlated with hepatocellular carcinoma (HCC) and to reveal a new potential target for early prediction, prevention, and diagnosis of HCC.

**Method:**

This study involved 96 cases with chronic hepatitis B (CHB), 96 cases with hepatitis B virus‐related liver cirrhosis (LC), 204 cases with HCC related to the hepatitis B virus, and 210 healthy controls. The genotype of rs8193 was determined using the restriction fragment length polymorphism method, while the genotypes of rs10836347 and rs13347 were determined by direct sequencing.

**Results:**

The results showed that patients with the CD44 rs13347 TT and T allele polymorphisms exhibited higher risks of LC than those carrying the CC genotype and C allele. The CD44 rs13347 CT and TT genotypes and T allele were significantly associated with an increased risk of HCC after adjusting for gender, age, smoking, and alcohol consumption (for CT: odds ratio [OR] = 1.626, 95% confidence interval [CI] = 1.057‐2.500, *P* = .027; for TT: OR = 1.965, 95% CI = 1.043‐3.702, *P* = .037; and for T: OR = 1.461, 95% CI = 1.091‐1.956, *P* = .011). In the rs13347 site of the female population, the CT and TT genotypes were related to the high occurrence of HCC. In the population aged ≥50 years, carriers of the CD44 rs13347 CT and TT alleles were more susceptible to HCC compared with CC carriers. Those who consumed alcohol who carried the rs10836347 CT genotype exhibited a risk factor for HCC.

**Conclusion:**

For the CD44 rs13347 site, mutations in the T allele might be a risk factor for HCC.

## 1 INTRODUCTION

Hepatocellular carcinoma (HCC) is one of the most frequent and fatal cancers. It is more common in less developed countries, but its incidence in the Western world is showing an alarming rise. The latest research reported 841 080 new cases of and 781 631 deaths from liver cancer in 2018.^1^ With its high morbidity and mortality rates of HCC, China accounted for about half of the total cases and deaths of HCC.^1^ In fact, Chen et al^2^ estimated that HCC had become the third major cause of cancer‐related death in China. Over the past few decades, surgical resection and a variety of comprehensive treatments have been used to treat HCC, but due to its high degree of malignancy, the prognosis is poor, and long‐term survival rates remain low. Therefore, the effective early detection, diagnosis, and treatment of HCC have become urgent.

The pathogenesis of a tumor is a multistep, multifactored, complicated process.^3^ Several studies have confirmed that environmental risk factors for HCC include infection with the hepatitis B virus (HBV) or hepatitis C virus (HCV), aflatoxin intake, alcohol use, and exposure to carcinogens.^4^, ^5^ However, under the same environmental conditions, some people develop HCC and some do not, suggesting that genetic factors influence HCC’s development.

CD44 is a major cell adhesion molecule of an extracellular matrix and has been observed in various cells and tissues. It is involved in many physiological processes, including cell proliferation, angiogenesis, invasion, and metastasis.^6^ The CD44 gene is located on chromosome 11 in humans and contains 20 exons, including 10 constant exons and 10 variant exons. Depending on whether the exons are involved in transcription and alternative splicing, CD44 is divided into a standard form (CD44s) and splice variant isoforms of the protein (CD44v).^7^ Several reports have documented that altered CD44 expression and the interaction of hyaluronan and CD44 may regulate cell growth, survival, invasion, and metastasis in various cancers.^8^, ^9^, ^10^

Chou et al^11^ identified that rs1425802, rs713330, rs11821102, rs10836347, and rs13347 polymorphisms of the CD44 gene were not correlated with risk of HCC. However, patients with the CD44 polymorphism rs187115 had higher risks of HCC. Similarly, Chen et al^12^ reported that rs187115 variant carriers with the G allele genotypes had an increased risk of HCC.

However, few studies have investigated the relationship between CD44 polymorphisms and HCC. Moreover, the regulation of CD44 expression in HCC is not completely understood. Therefore, this study thoroughly investigated the association between CD44 polymorphisms to elucidate a more exact relationship between CD44 polymorphisms and the risk of HCC. The study further tried to reveal a new potential target for the early prediction, prevention, and diagnosis of HCC.

## 2 METHODS

### 2.1 Study population

This study involved 96 cases with chronic hepatitis B (CHB; 77 males and 19 females), 96 cases with HBV‐related liver cirrhosis (LC; 75 males and 21 females), 204 cases with HBV‐related HCC (169 males and 35 females), and 210 healthy controls (153 males and 57 females). Data from patients with HBV‐infected disease were collected from the First Affiliated Hospital of Guangxi Medical University between September 2014 and June 2015. The healthy control subjects were selected from patients who visited the health center of the First Affiliated Hospital of Guangxi Medical University for routine physical examinations between September 2014 and June 2015.

The criteria for CHB included having abnormal levels of alanine aminotransferase (ALT) and aspartate aminotransferase (AST) and simultaneously being persistent HBV surface antigen‐positive for more than half a year. HBV‐related LC was defined as being HBV surface antigen‐positive, in which the liver pathology showed clinical manifestations of portal hypertension and in which ultrasonography showed evidence of cirrhosis. HCC patients were confirmed by imaging examinations, including ultrasonography, magnetic resonance imaging, or computed tomography. Additionally, the a‐fetoprotein (AFP) elevation of these patients was higher than the cutoff value of >400 ng/mL.

Healthy controls were included who had previously been examined and had no tumor, who were HBsAg‐negative, and who had normal liver function and other laboratory tests. They had no family history of tumors or major disease. Nonsmokers and nondrinkers were defined as those who had never smoked or consumed alcohol. Questionnaires included demographic information such as sex, age, and smoking and drinking habits, and informed consent was collected from each enrolled participant. The study approval was obtained from the ethics committee of the First Affiliated Hospital of Guangxi Medical University.

### 2.2 Selection of CD44 polymorphisms

A total of three single nucleotide polymorphisms (SNPs) in CD44 were selected for this study. Rs8193 was selected since the gene polymorphisms of this SNP have been found to be associated with colon cancer recurrence and gastric cancer.^8^, ^9^ The rs10836347 and rs13347 SNPs located in the 3′ untranslated region (3′ UTR) and the T allele mutations in the CD44 rs13347 site were chosen because they affect gene transcriptional activity.^10^

### 2.3 CD44 genotyping

Genomic DNA was isolated from fresh blood samples from each patient using the QIAamp DNA Blood Mini Kit according to the manufacturer's instructions. The upstream and downstream primers of rs8193, rs10836347, and rs13347 were designed and synthesized by the Shanghai Sangon Biotech Company. Since rs10836347 and rs13347 were close to each other, the two sites were combined to design primers, and the genotypes of the two sites could be determined by sequencing. The genotyping of rs8193 was performed using the restriction fragment length polymorphism method. The sequence of primers used in the polymerase chain reaction (PCR) and the essential reaction conditions are shown in Table 1.

**Table 1:** Primer sequence and the reaction condition for genotyping CD44 polymorphisms

Polymorphism	Primer sequence	Annealing temperature (°C)	Restriction enzyme	Product size (bp)
rs8193	F: 5′‐ CCCCACCAGCTAAGGACATT ‐3'	64	BseMI	CC: 97 + 391 TT: 488
	R: 5′‐ AGTGGGAACTCAGGGTCTCT ‐3′			CT: 97 + 391 + 488
rs10836347 and rs13347	F: 5′‐ AGAGATTTCCTGGGTCTGCC ‐3′	60		
	R: 5′‐ GACTTAGCCCAGGTCACCT ‐3′			

### 2.4 Statistical analysis

The genotype and allele frequencies of SNPs in the four groups were calculated using the direct calculation method. Demographic and clinical data among the groups were analyzed using the ANOVA method. The SNPs were analyzed for deviation from the Hardy‐Weinberg equilibrium (HWE) using the chi‐square test. Binary logistic regression was performed to determine the odds ratio (OR), and four factors (gender, age, smoking, and alcohol consumption) were adjusted. Stratified analyses were assessed according to gender, age, smoking, and alcohol consumption. All data were analyzed with SPSS 13.0. When the *P* value was less than .05, the results were considered significant.

## 3 RESULTS

### 3.1 Characteristics of study populations

The features of the three disease groups and controls included in this study are listed in Table 2. Significant statistical difference was found regarding the age (all *P* < .001), while the proportions of the sexes in the four groups were consistent (*P* = .167, .327 and .053). The smoking and drinking status of the patients was taken into account with respect to its influence on the association between CD44 polymorphisms and HCC. For smoking, a significant difference was found between controls and HBV‐related liver cirrhosis (*P* = .005). For drinking, a significant differences were found between controls and chronic hepatitis B (*P* = .036) and HCC (*P* = .009).

**Table 2:** Characteristics of study subjects

Groups	Healthy controls （HC）(n = 210)	Chronic HBV patients (n = 96)	*P*	HBV‐related LC (n = 96)	*P*	HBV‐related HCC patients (n = 204)	*P*
Age (y) (Mean ± SD)	46.6 ± 6.9	37.8 ± 12.4	<.001	49.9 ± 11.1	<.001	49.2 ± 11.1	<.001
Gender, n（%）							
Male	153 (72.9)	77 (80.3)	.167	75 (78.1)	.327	165 (80.8)	.053
Female	57 (27.1)	19 (19.7)		21 (21.9)		39 (19.2)	
Smoking status, n（%）							
Yes	64 (30.5)	38 (39.5)	.117	45 (46.8)	.005	73 (35.8)	.251
No	146 (69.5)	58 (60.5)		51 (53.2)		131 (64.2)	
Drinking, n（%）							
Yes	56 (26.7)	37 (38.5)	.036	35 (36.4)	.082	79 (38.7)	.009
No	154 (73.3)	59 (61.5)		61 (63.6)		125 (61.3)	

Tests for the HWE were performed separately for all SNPs in the healthy controls and then for the three disease groups; the observed genotype frequencies were both in agreement with the HWE (all *P* > .05).

### 3.2 Alleles and genotype distributions of CD44 polymorphisms

The allele and genotype distributions of CD44 rs8193, rs10836347, and rs13347 among the disease groups and the controls are shown in Table 3. Binary logistic regression analysis for the CD44 rs8193 polymorphism (after adjusting for sex, age, smoking, and alcohol consumption) revealed that there were no statistically significant differences in allele and genotype distribution between cases and controls. The results showed that the CD44 rs8193 polymorphisms had no relationship with the risk of CHB, LC, or HCC in any genetic models. Additionally, when the rs10836347 site was analyzed, no significant difference was found between the controls and the cases (Table 3).

**Table 3:** Genotype distributions and allele frequencies of CD44 polymorphisms between disease groups and controls

Polymorphisms	HC (n = 210)	CHB (n = 96)	LC (n = 96)	HCC (n = 204)	CHB vs HC		LC vs HC		HCC vs HC	
					OR (95% CI)	*P*	OR (95% CI)	*P*	OR (95% CI)	*P*
rs8193										
Genotypes										
CC	66 (31.4)	37 (38.5)	36 (37.5)	61 (29.9)	1.00^ref^		1.00^ref^		1.00^ref^	
CT	103 (49.1)	44 (45.8)	46 (47.9)	112 (54.9)	0.649 (0.351‐1.199)	.167	0.906 (0.520‐1.578)	.728	1.173 (0.748‐1.838)	.488
TT	41 (19.5)	15 (15.7)	14 (14.6)	31 (15.2)	0.740 (0.328‐1.670)	.469	0.685 (0.322‐1.458)	.327	0.809 (0.446‐1.467)	.485
Allele										
C	235 (55.9)	118(61.4)	118(61.4)	234 (57.4)	1.00^ref^		1.00^ref^		1.00^ref^	
T	185 (44.1)	74 (38.6)	74 (38.6)	174 (42.6)	0.821 (0.553‐1.219)	.328	0.843 (0.588‐1.207)	.350	0.940 (0.710‐1.246)	.667
Dominant model										
CC	66 (31.4)	37 (38.5)	36 (37.5)	61 (29.9)	1.00^ref^		1.00^ref^		1.00^ref^	
CT + TT	144 (68.6)	59 (61.5)	60 (62.5)	143 (70.1)	0.672 (0.377‐1.197)	.177	0.843 (0.499‐1.424)	.523	1.068 (0.696‐1.637)	.763
Recessive model										
CC + CT	169 (80.5)	81 (84.3)	82 (85.4)	173 (84.8)	1.00^ref^		1.00^ref^		1.00^ref^	
TT	41 (19.5)	15 (15.7)	14 (14.6)	31 (15.2)	0.955 (0.459‐1.986)	.901	0.726 (0.366‐1.438)	.358	0.733 (0.433‐1.239)	.246
rs10836347										
Genotypes										
CC	98 (46.7)	40 (41.7)	36 (37.5)	73 (35.8)	1.00^ref^		1.00^ref^		1.00^ref^	
CT	90 (42.9)	48 (50)	40 (41.7)	99 (48.5)	0.304 (0.089‐1.039)	.057	1.306 (0.619‐2.757)	.484	1.406 (0.784‐2.251)	.253
TT	22 (10.4)	8 (8.3)	20 (20.8)	32 (15.7)	NA		NA		NA	
Allele										
C	286 (68.1)	128(66.7)	112(58.3)	245 (60.0)	1.00^ref^		1.00^ref^		1.00^ref^	
T	134 (31.9)	64 (33.3)	80 (41.7)	163 (40.0)	0.318 (0.095‐1.065)	.063	1.282 (0.624‐2.632)	.499	1.372 (0.782‐2.409)	.271
rs13347										
Genotypes										
CC	98 (46.7)	40 (41.7)	36 (37.5)	73 (35.8)	1.00^ref^		1.00^ref^		1.00^ref^	
CT	90 (42.9)	48 (50)	40 (41.7)	99 (48.5)	1.225 (0.688‐2.183)	.490	1.282 (0.737‐2.230)	.379	1.626 (1.057‐2.500)	.027
TT	22 (10.4)	8 (8.3)	20 (20.8)	32 (15.7)	1.033 (0.382‐2.795)	.948	2.601 (1.242‐5.447)	.011	1.965 (1.043‐3.702)	.037
Allele										
C	286 (68.1)	128(66.7)	112(58.3)	245 (60.0)	1.00^ref^		1.00^ref^		1.00^ref^	
T	134 (31.9)	64 (33.3)	80 (41.7)	163 (40.0)	1.085 (0.720‐1.634)	.697	1.588 (1.102‐2.288)	.013	1.461 (1.091‐1.956)	.011
Dominant model										
CC	98 (46.7)	40 (41.7)	36 (37.5)	73 (35.8)	1.00^ref^		1.00^ref^		1.00^ref^	
CT + TT	112 (53.3)	56 (58.3)	60 (62.5)	131 (64.2)	1.192 (0.683‐2.079)	.536	1.551 (0.930‐2.587)	.093	1.697 (1.130‐2.549)	.011
Recessive model										
CC + CT	188 (89.4)	88 (91.7)	76 (79.2)	172 (84.3)	1.00^ref^		1.00^ref^		1.00^ref^	
TT	22 (10.4)	8 (8.3)	20 (20.8)	32 (15.7)	0.927(0.361‐2.381)	.874	2.304(1.162‐4.568)	.017	1.524 (0.844‐2.752)	.162

Abbreviations: CHB, chronic HBV; HC, healthy controls; HCC, hepatocellular carcinoma; LC, liver cirrhosis.

Nonsignificant associations with CHB risk were suggested for CD44 rs13347 when the healthy controls were compared with the CHB cases (Table 3). However, patients with the CD44 rs13347 TT and T allele polymorphisms exhibited 2.601‐fold (95% CI = 1.242‐5.447, *P* = .011) and 1.588‐fold (95% CI = 1.102‐2.288, *P* = .013) higher risks of LC, respectively, than those carrying the CC genotype and C allele. Similar results were found in the recessive analytic model; carriers of the TT genotype for CD44 rs13347 had a twofold increased risk of LC (95% CI = 1.162‐4.568, *P* = .017). After adjustment for the four risk factors, a higher risk of HCC was experienced by carriers of the CD44 rs13347 heterozygous variant (CT; OR = 1.626, 95% CI = 1.057‐2.500, *P* = .027), the homozygote variant (TT) genotype (OR = 1.965, 95% CI = 1.043‐3.702, *P* = .037), and the T allele (OR = 1.461, 95% CI = 1.091‐1.956, *P* = .011) when compared to the healthy controls. In the dominant analytic model, significant effects were found, with OR = 1.697 (95% CI = 1.130‐2.549, *P* = .011).

### 3.3 Stratification of CD44 polymorphisms according to sex, age, smoking, and alcohol consumption

The risk of HCC related to the CD44 rs8193, rs10836347, and rs13347 genotypes was further analyzed with stratification by sex, age, smoking, and alcohol consumption. No evidence of CD44 polymorphisms with respect to HCC susceptibility was found in the male population. In the female population, the risk of HCC increased for the rs13347 site when the individual carried the CT and TT genotypes, with OR = 2.675 (95% CI = 1.004‐7.1329, *P* = .049) and OR = 5.992 (95% CI = 1.083‐33.164, *P* = .040), respectively (Table 4). When the population was divided into <50 and ≥50 age‐groups, the results showed that CD44 polymorphisms were not associated with HCC susceptibility in the <50 population. However, in the ≥50 population, CT and TT genotype carriers (OR = 2.517, 95% CI = 1.113‐4.181, *P* = .023; and OR = 3.405, 95% CI = 1.263‐9.178, *P* = .015, respectively) had an increased risk of HCC versus CC genotype carriers for the rs13347 site (Table 5). Whether the person was a smoker or nonsmoker, no evidence of an association between CD44 polymorphisms and HCC susceptibility was found (Table 6). Table 7 shows that drinkers with an rs10836347 CT genotype had a higher risk of HCC (OR = 4.552, 95% CI = 1.205‐17.192, *P* = .025). Meanwhile, the rs13347 TT genotype was significantly related to HCC susceptibility (OR = 4.549, 95% CI = 1.244‐16.635, *P* = .022; Table 7).

**Table 4:** Stratified effects of CD44 polymorphisms on HCC risk estimated by sex

	Male				Female			
	HCC	HC	OR (95% CI)	*P*	HCC	HC	OR (95% CI)	*P*
rs8193								
CC	53	51	11.00^ref^		8	15	1.00^ref^	
CT	84	75	0.987 (0.594‐1.639)	.958	28	28	2.340 (0.802‐6.824)	.120
TT	28	27	0.988 (0.504‐1.936)	.973	3	14	0.616 (0.127‐2.985)	.547
rs10836347								
CC	142	135	11.00^ref^		31	51	1.00^ref^	
CT	23	18	1.168 (0.594‐2.299)	.652	8	6	2.100 (0.629‐7.017)	.228
TT	0	0	NA		0	0		
rs13347								
CC	63	71	11.00^ref^		10	27	1.00^ref^	
CT	76	63	1.460 (0.892‐2.388)	.132	23	27	2.675 (1.004‐7.132)	.049
TT	26	19	1.635 (0.812‐3.292)	.169	6	3	5.992 (1.083‐33.164)	.040

**Table 5:** Stratified effects of CD44 polymorphisms on HCC risk estimated by age

	Age <50 y				Age ≥50 y			
	HCC	HC	OR (95% CI)	*P*	HCC	HC	OR (95% CI)	*P*
rs8193								
CC	29	40	11.00^ref^		32	26	1.00^ref^	
CT	63	64	1.534 (0.820‐2.871)	.181	49	39	1.000 (0.510‐1.961)	1.000
TT	17	25	1.104 (0.487‐2.501)	.813	14	16	0.730 (0.298‐1.789)	.491
rs10836347								
CC	92	113	11.00^ref^		81	73	1.00^ref^	
CT	17	16	1.395 (0.644‐3.022)	.398	14	8	1.678 (0.658‐4.283)	.279
TT	0	0	NA		0	0	NA	
rs13347								
CC	39	51	11.00^ref^		34	47	1.00^ref^	
CT	55	63	1.143 (0.642‐2.038)	.649	44	27	2.157 (1.113‐4.181)	.023
TT	15	15	1.201 (0.507‐2.845)	.677	17	7	3.405 (1.263‐9.178)	.015

**Table 6:** Stratified effects of CD44 polymorphisms on HCC risk estimated by smoking

	Smoker				Nonsmoker			
	HCC	HC	OR (95% CI)	*P*	HCC	HC	OR (95% CI)	*P*
rs8193								
CC	21	19	11.00^ref^		40	47	1.00^ref^	
CT	43	34	1.180 (0.529‐2.630)	.686	69	69	1.234 (0.708‐2.152)	.458
TT	9	11	0.814 (0.253‐2.621)	.730	22	30	0.961 (0.417‐1.962)	.914
rs10836347								
CC	61	58	11.00^ref^		112	128	1.00^ref^	
CT	12	6	2.135 (0.729‐6.259)	.167	19	18	1.141 (0.559‐2.327)	.718
TT	0	0	NA		0	0	NA	
rs13347								
CC	26	29	11.00^ref^		47	69	1.00^ref^	
CT	35	28	1.606 (0.746‐3.457)	.225	64	62	1.659 (0.975‐2.822)	.062
TT	12	7	2.071 (0.684‐6.269)	.198	20	15	1.947 (0.887‐4.275)	.097

**Table 7:** Stratified effects of CD44 polymorphisms on HCC risk estimated by alcohol consumption

	Drinker				Nondrinker			
	HCC	HC	OR (95% CI)	*P*	HCC	HC	OR (95% CI)	*P*
rs8193								
CC	21	16	11.00^ref^		40	50	1.00^ref^	
CT	45	29	1.387 (0.592‐3.246)	.451	67	74	1.153 (0.668‐1.989)	.609
TT	13	11	1.219 (0.397‐3.736)	.730	18	30	0.844 (0.404‐1.763)	.653
rs10836347								
CC	63	53	11.00^ref^		110	133	1.00^ref^	
CT	16	3	4.552 (1.205‐17.192)	.025	15	21	0.835 (0.406‐1.719)	.625
TT	0	0	NA		0	0	NA	
rs13347								
CC	28	28	11.00^ref^		45	70	1.00^ref^	
CT	36	24	1.710 (0.782‐3.741)	.179	63	66	1.590 (0.939‐2.690)	.084
TT	15	4	4.549 (1.244‐16.635)	.022	17	18	1.497 (0.689‐3.253)	.308

### 3.4 Haplotype analyses of CD44 SNPs and HCC risk

Haplotype analyses were further performed in HCC patients and healthy controls using the SHEsis software. Four possible haplotypes (CCC, CCT, TCC, and TCT) were derived from the observed genotypes, and their distributions in both groups are shown in Table 8. The results showed that haplotypes were not associated with HCC risk (all *P* > .05).

**Table 8:** Haplotype frequencies of CD44 polymorphisms

Haplotypes	Case (2n = 404, %)	Controls (2n = 420, %)	*P*	OR	95% CI
CCC	130 (0.320)	145 (0.346)	.534	0.911	0.678‐1.223
CCT	91 (0.222)	73 (0.174)	.057	1.399	0.989‐1.979
TCC	97 (0.238)	125 (0.279)	.080	0.756	0.553‐1.035
TCT	59 (0.144)	53 (0.126)	.374	1.200	0.803‐1.793

## 4 DISCUSSION

CD44 is a multifunctional, transmembrane glycoprotein that was first defined as a lymphocyte homing receptor expressed in embryonic stem cells, hematopoietic stem cells, and tumor stem cells. CD44 is related to a series of basic biological processes, including lymphocyte homing, cell migration, inflammation, wound healing, embryonic development, and cell apoptosis.^13^ At the same time, CD44 also mediates signaling pathways of tumor differentiation, invasion, and metastasis and influences tumor development.^14^

Polymorphism is defined as an instance of the same gene with different frequencies of distribution in different populations; this is the most fundamental cause of individual differences, and it also determines individuals’ different susceptibilities to disease. SNPs were reported to be involved in HBV‐related CHB, LC, and HCC. Wand et al^15^ observed that FABP1 rs1545224 AG and AA genotypes might increase HCC risk in LC patients when compared to the GG genotype. Results from a meta‐analysis including 3217 cases and 4163 controls showed that miR‐146a rs291016 was associated with increasing hepatitis virus—related HCC risk in overall analysis. Conversely, miR‐196a2 rs11614913 was found to decrease hepatitis virus—related HCC risk in overall analysis.^16^

Over the past decade, many studies have been conducted to examine the contribution of CD44 polymorphisms to the risk of cancer. Zhou et al 17 used direct nucleotide sequencing analysis SNP CD44 Ex2 + 14 A > G, located in the intron 1 region, and demonstrated for the first time that +14GG genotype carriers are more susceptible to breast cancer than +14A/G genotype carriers in both Caucasian and African American women. The variant genotype is significantly associated with a larger tumor burden, more regional lymph node metastasis, and higher cancer recurrence. The results from the peripheral blood experiment showed that CD44 cells were significantly reduced in healthy human GG carriers, which indicated that gene polymorphism may influence the shearing and splicing of CD44, leading to a decrease in the CD44 protein and affecting the body's susceptibility to breast cancer risk.^17^

For the five SNPs of CD44 (rs10836347C > T, rs13347C > T, rs1425802A > G, rs11821102G > A, and rs713330T > C), Jiang et al^10^ found that individuals in Suzhou with the rs13347C > T CT and TT genotypes had a higher risk of breast cancer than CC carriers (for CT: OR = 1.69; for TT: OR = 2.22), and the same results have also been confirmed in the southern Chinese populations. However, Tulsyan et al^18^ did not find an association between the polymorphisms of rs13347C > T and the risk of breast cancer in India.

The results from a study by Liu et al^19^ showed that rs187115 sites of the G allele significantly increased the risk of non‐small‐cell lung cancer (NSCLC) compared with the AA genotype. They also found that the tumor grade of AG and GG gene carriers was higher and were more prone to bone metastases. However, the researchers found no association between rs13347 and NSCLC risk.

Other studies have also demonstrated that the CD44 gene has a role in the regulation of tumor metastasis and that a high expression of CD44 is usually present in metastatic tumors, especially in tumors with higher bone metastasis.^20^, ^21^ These results provide a good biological basis for the study of genetic susceptibility to CD44 and cancer.

A study in Taiwan showed that individuals with the AG, GG, and AG + GG genotypes of the SNP rs187115 of CD44 had a significantly higher risk of HCC than those with the AA genotype. The other five SNPs (rs1425802, rs11821102, rs10836347, rs13347, and rs713330) did not contribute to the risk of HCC.^11^ However, there have been no further reports of CD44 and its related susceptibility to HCC to date on.

This was the first study to report on the association between the CD44 polymorphisms rs8193, rs10836347, and rs13347 and susceptibility to HBV‐related liver disease. The results revealed that none of the rs8193 and rs10836347 SNPs of CD44 was associated with susceptibility to HCC. Data from the literature have indicated that the CD44 gene rs8193 is associated with tumor recurrence; the median tumor recurrence time for the T allele is 9.4 years, while that for the wild homozygous CC genotype is 5.4 years (hazard ratio (HR) = 0.51, 95% CI = 0.35‐0.93, *P* = .022).^8^ A similar result was found in a gastric cancer study conducted on the Chinese population. The data showed that, for rs8193, the TT genotype carrier had smaller tumors without serosal invasion compared to the CC genotype. This indicated that the T rs8193 gene might be a protective factor in gastric cancer and that it might be used to monitor tumor growth and progression.^9^ The reason for these inconsistent results may be that the rs8193 CD44 polymorphism plays different roles in different tumors.

The human CD44 rs13347 site is located in the 3' UTR region; it has been reported that the transcriptional activity of the rs13347 T allele is higher than that of the C allele. Immunohistochemistry and Western blotting results also showed that the CD44 expression was significantly higher in T allele carriers than in C allele carriers.^22^ Similar results were confirmed in a breast cancer study in which Jiang et al^10^ showed that breast cancer patients with CT and TT had higher levels of CD44 than CC genotype carriers, while the 5‐year survival rate of CT + TT patients was significantly lower.

The T allele mutations in the CD44 rs13347 site affect gene transcriptional activity, which leads to changes in protein levels and contributes to the risk of cancer. This present study demonstrated that, for the rs13347 site, TT and T alleles yielded a 2.601‐fold and 1.588‐fold trend for increased risk of LC, respectively. A further recessive analytic model showed that the TT genotype for CD44 rs13347 seems to carry an increased risk of LC (OR = 2.304, 95% CI = 1.162‐4.568, *P* = .017). Similarly, the CT and TT genotypes and the T allele were significantly associated with an increased risk of HCC after the adjustment for gender, age, smoking, and alcohol consumption (for CT: OR = 1.626; for TT: OR = 1.965; and for T: OR = 1.461). In the dominant analytic model, OR = 1.697 for HCC when the genotype at rs13347 was CT + TT. The results of the current study showed that the carriage of the allele T at rs13347 played a role in the risk for development of LC and HCC. Therefore, the study hypothesized that the link between the CD44 polymorphisms and HCC risk could be achieved by altering the protein expression of immune cells. However, the potential mechanism should be further investigated.

A large number of studies have demonstrated that the incidence of HCC in men is significantly higher than that in women. In different populations, the prevalence of HCC in men and women can be from 2:1 to 4:1.^23^, ^24^ In this study, a stratified analysis was performed by sex, and a significantly increased risk of HCC among female patients was observed. For rs13347, the CT genotype might have increased the risk of HCC by 2.675 times, and the TT genotype was correlated with an increased risk of HCC of 5.922 times. In breast cancer, data from the literature have indicated that T alleles at rs13347 significantly increase the risk of breast cancer.^10^ Therefore, it was hypothesized that mutations in rs13347 of the CD44 gene in female populations might be involved in the development of cancer.

Data from the literature have also indicated that excessive alcohol consumption is an important risk factor for HCC; in the present study, the stratified analysis found that the rs10836347 CT genotype and the rs13347 TT genotype were correlated with a higher risk of HCC in those who consumed alcohol, suggesting that those two genotypes might be risk factors for HCC. Interactions between osteopontin (OPN) and CD44 have been reported to inhibit the expression of the IL‐10 Th2 cytokine; additionally, these interactions have been shown to be involved in inflammatory response.^25^ Long‐term alcohol usage may induce liver cell injury and cause a sustained immune response, which might explain the possible role of the CD44 polymorphisms in HCC.^25^

However, a few limitations remained in this study. First, the total sample size was relatively small, especially for the HBV‐related LC subgroup. Second, only three SNPs were selected; more SNPs should be studied. Third, this case‐control study only involved the Chinese population of the Guangxi province. Therefore, further well‐designed studies are demanded from other regions of China. Finally, a gene expression functional assay was not taken; this should be taken in the future.

## 5 CONCLUSION

In conclusion, data from this study indicated that CD44 rs13347 polymorphisms were associated with the risk of HCC, and mutations in the T allele might be a risk factor for HCC.

## CONFLICT OF INTEREST

The authors declare that they have no competing interests.

## AUTHOR CONTRIBUTIONS

XQ and SL conceived and designed the experiments. YD and ZJC carried out the experiments. FL and QTH analyzed the data, and YD, SYC, and YFD wrote the article.

## ETHICS APPROVAL AND CONSENT TO PARTICIPATE

This study was approved by the ethics committee of the First Affiliated Hospital of Guangxi Medical University, and all participants provided written informed consent.

## CONSENT FOR PUBLICATION

Not applicable.

## Data Availability

All data generated or analyzed during this study are included in this published article.
